# Supplementary material for: A high throughput method for egg size measurement in Drosophila
Source: Sci Rep. 2023 Mar 7;13:3791. doi: 10.1038/s41598-023-30472-8 (PMC9992389; doi:10.1038/s41598-023-30472-8)
Supplement: Supplementary file 1 — Supplementary Information. [file 41598_2023_30472_MOESM1_ESM.docx]

**Supplementary figures and Tables**

**Fig. S1** Time of flight (TOF), the measured time an object blocks the light, is used to estimate the object’s size. *Drosophila* eggs are washed and dispensed in 1x PBS buffer and run on large particle flow cytometry. TOF and extinction are measured (A) and TOF is converted to size (μm) using the TOF data of reference beads. Based on the size distribution, user-defined sorting gates are set up (depicted as dotted lines in (A), these gates correspond to size 200-900 μm with intervals of 100μm), and the required number of eggs from each gate can be sorted. Red, yellow and green bars in (B) show the number of sorted eggs in these 3 size gates in (A). **
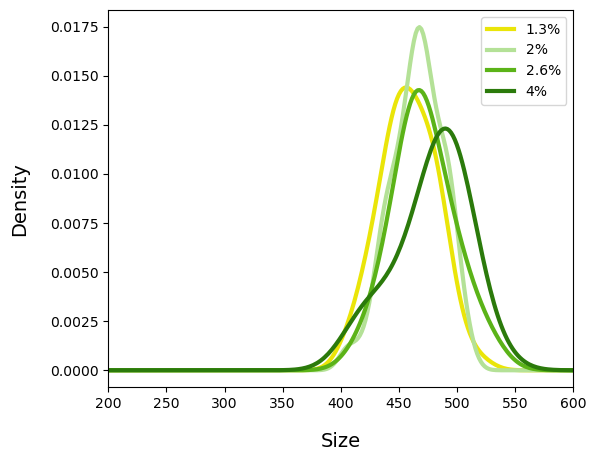
**

**Fig. S2** Distribution of the egg length estimated using different concentrations of methyl cellulose (MC) in 1x PBS as sheath buffer. As the concentration of MC increases the sheath buffer becomes more viscous which will reduce the flow rate. The slower flow rate facilitates the alignment of objects along the long axis while passing through the laser path. However, the increased viscosity of the sheath buffer dramatically reduces the speed of analysis. The same sample has been used for all runs shown in the plot.


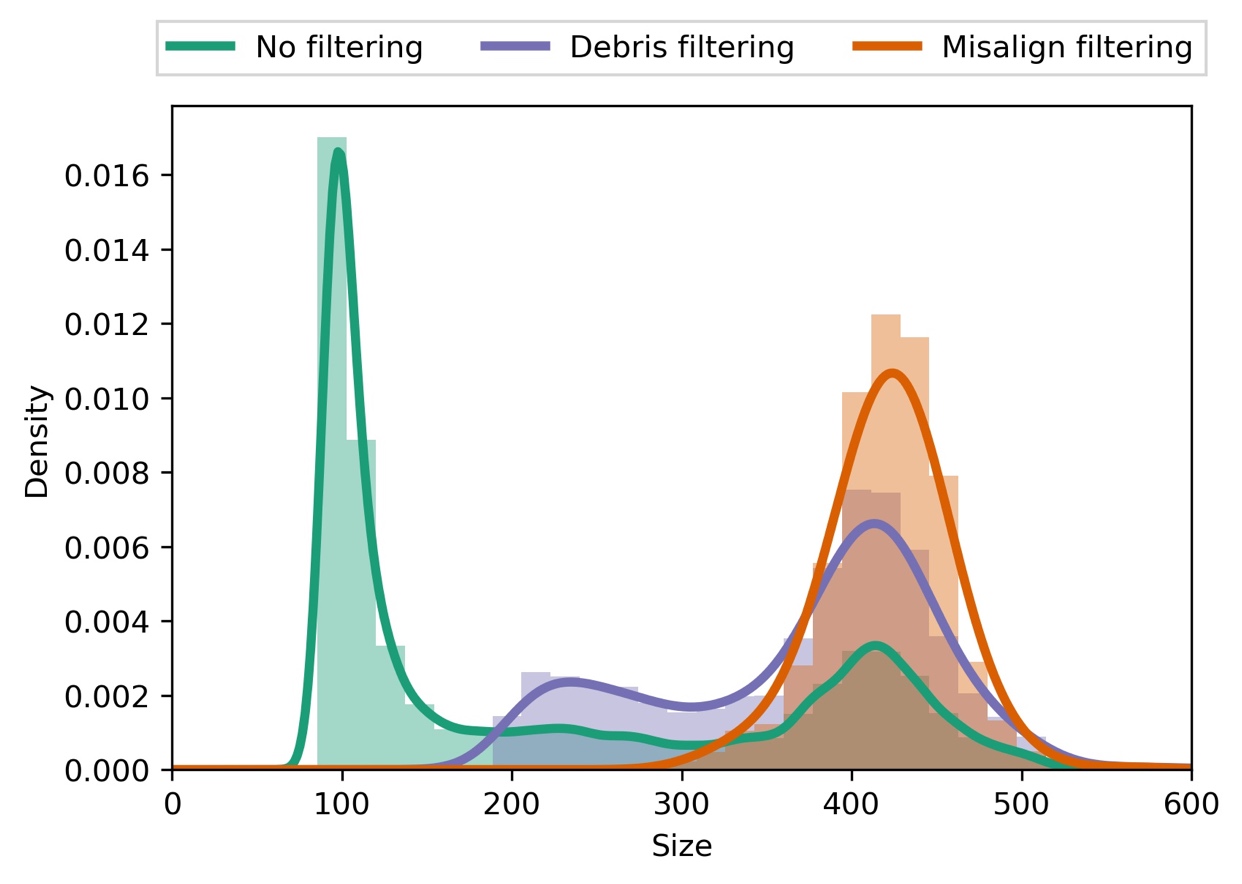


**Fig. S3** Distribution of the egg length in sample Dsim196 before and after in silico filtering of debris and misaligned objects. The initial size distribution is bimodal (No filtering). The 1^st^ mode corresponds to debris and yeast particles, and ‘Debris filtering’ remove these small objects. Further filtering steps based on EI and W/L parameters (Misalign filtering) removed the misaligned eggs. Size is in µm units. The statistics of egg length distribution is presented in Table S1 and S2.

**Table S1** The statistics of egg length distribution. Size threshold is used to remove 1^st^ peak containing egg debris from the 2^nd^ mode in the first filtering step. Dsim#, #: stock number (*D. simulans*), Lausanne5 (*D. melanogaster*), Dere01 (D*. erecta*), Dsan01 (D*. santomea*) and Dmau151 (*D. mauritiana*). Except for *D. simulans*, other inbred lines are obtained from Bloomington stock center. *D. simulans* inbred lines are provded by Sara Signor (North Dakota State University).

| Sample | 1st peak mode (μm) | size threshold (μm) | 2nd peak mode (μm) |
| --- | --- | --- | --- |
| Dsim1 | 94.903 | 212.212 | 449.078 |
| Dsim4 | 92.209 | 176.977 | 459.851 |
| Dsim5 | 92.209 | 196.196 | 427.531 |
| Dsim11 | 92.209 | 177.778 | 426.184 |
| Dsim75 | 92.209 | 207.407 | 450.425 |
| Dsim90 | 92.209 | 173.774 | 427.531 |
| Dsim91 | 92.209 | 92.893 | 93.556 |
| Dsim146 | 92.209 | 180.981 | 450.425 |
| Dsim166 | 92.209 | 198.599 | 436.958 |
| Dsim185 | 92.209 | 201.802 | 426.184 |
| Dsim196 | 92.209 | 194.595 | 423.491 |
| Dsim237 | 92.209 | 182.583 | 431.571 |
| Lausanne5 | 92.209 | 146.547 | 420.798 |
| Dere01 | 92.209 | 207.407 | 431.571 |
| Dsan01 | 92.209 | 211.411 | 384.438 |
| Dmau151 | 92.209 | 187.387 | 455.811 |

**Table S2** The statistics of egg length distribution filtered using sample-specific threshold. Each dataset is filtered as described in Fig. 1. The 1^st^ filtering step is based on the size threshold in Table S1. The filtered objects at this step are mostly egg debris. # nonegg: the number of small objects filtered in the first filtering stage. The mode, mean and median of these objects are Mode nonegg, Mean nonegg, and Median nonegg respectively. The second filtering step involved removing the misaligned eggs. The number, mean and median of these misaligned eggs are shown as #misaligned egg, Mean misaligned egg and Median misaligned egg. The last filtering step is to remove the objects in the first peak of distribution after removal of the misaligned eggs. Size threshold (μm) is used for this filtering step. #egg, Mode egg, Mean egg and Median egg are the number, mode, mean and median of the eggs in the final dataset. Dsim#, #: stock number (*D. simulans*), Lausanne5 (*D. melanogaster*), Dere01 (*D. erecta*), Dsan01 (*D. santomea*) and Dmau151 (*D. mauritiana*). Except for *D. simulans*, other inbred lines are obtained from Bloomington stock center. *D. simulans* inbred lines are provded by Sara Signor (North Dakota State University).

| Sample | size threshold (μm) | #egg | #nonegg | #misaligned egg | Mode nonegg | Mean nonegg | Median nonegg | Mode egg | Mean egg | Median egg | Mean misaligned egg | Median misaligned egg |
| --- | --- | --- | --- | --- | --- | --- | --- | --- | --- | --- | --- | --- |
| Dsim1 | 269.069 | 361 | 2710 | 849 | 94.903 | 110.546 | 102.983 | 440.998 | 438.457 | 442.345 | 382.667 | 391.171 |
| Dsim4 | 283.483 | 1980 | 6707 | 4572 | 92.209 | 111.721 | 105.676 | 458.505 | 453.336 | 457.158 | 373.373 | 387.131 |
| Dsim5 | 248.248 | 265 | 917 | 699 | 92.209 | 108.428 | 102.983 | 427.531 | 443.051 | 447.731 | 384.705 | 389.824 |
| Dsim11 | 241.041 | 458 | 1530 | 1211 | 92.209 | 108.814 | 102.983 | 420.798 | 426.752 | 431.571 | 377.862 | 385.784 |
| Dsim75 | 263.463 | 955 | 3678 | 2396 | 92.209 | 109.350 | 101.636 | 457.158 | 454.088 | 457.158 | 393.318 | 399.251 |
| Dsim90 | 221.822 | 941 | 3075 | 2119 | 92.209 | 106.286 | 100.289 | 427.531 | 434.046 | 438.304 | 384.049 | 397.904 |
| Dsim91 | 217.818 | 1913 | 1707 | 14055 | 92.209 | 92.209 | 92.209 | 438.304 | 416.989 | 426.184 | 128.336 | 102.983 |
| Dsim146 | 256.256 | 1229 | 6138 | 2957 | 92.209 | 106.108 | 100.289 | 455.811 | 454.377 | 459.851 | 389.055 | 404.638 |
| Dsim166 | 274.675 | 906 | 4005 | 2263 | 92.209 | 110.152 | 102.983 | 453.118 | 461.060 | 462.545 | 391.385 | 389.824 |
| Dsim185 | 249.049 | 1535 | 12413 | 3878 | 92.209 | 110.019 | 101.636 | 443.691 | 437.533 | 440.998 | 376.696 | 385.784 |
| Dsim196 | 298.699 | 1630 | 10442 | 6186 | 92.209 | 111.171 | 101.636 | 423.491 | 421.893 | 422.144 | 362.492 | 380.398 |
| Dsim237 | 237.838 | 725 | 3422 | 2147 | 92.209 | 106.550 | 100.289 | 427.531 | 429.827 | 431.571 | 396.969 | 388.478 |
| Lausanne5 | 163.363 | 689 | 4069 | 2401 | 92.209 | 107.065 | 102.983 | 420.798 | 363.069 | 404.638 | 312.924 | 315.757 |
| Dere01 | 221.021 | 2056 | 1453 | 4757 | 92.209 | 114.704 | 104.329 | 431.571 | 433.829 | 434.264 | 392.111 | 403.291 |
| Dsan01 | 317.117 | 250 | 2169 | 929 | 92.209 | 113.170 | 104.329 | 403.291 | 411.872 | 410.024 | 364.281 | 365.584 |
| Dmau151 | 277.878 | 3079 | 15325 | 6214 | 92.209 | 108.169 | 101.636 | 466.585 | 464.235 | 465.238 | 381.830 | 388.478 |
